# Crowded out

**Published:** 1888-04

**Authors:** 


### CROWDED OUT.

In consequence of the great amount of matter for the "Current News" department that came in at the last moment, some of the editorials, which were all in type, have been obliged to give way. The leading one, on "Anæsthesia and Anæsthetics," was the first to be superseded, and some shorter ones took their place with it upon the standing galley. They will perhaps keep a month, and if not, they can very well be spared altogether.

